# Survival prognostic impact of post-surgical complications in gastric cancer patients after neoadjuvant chemotherapy: a single western center analysis

**DOI:** 10.1007/s13304-025-02370-3

**Published:** 2025-08-19

**Authors:** Nicola Natalizi, Luigina Graziosi, Fabiola Di Schiena, Annibale Donini

**Affiliations:** Digestive Surgery, University of Perugia, Perugia, Italy

**Keywords:** gastric cancer, neoadjuvant chemotherapy, postoperative complications, prognosis

## Abstract

**Introduction:**

It’s well known that postoperative complications (POC) negatively invalidate survival of patients who underwent surgery for gastric cancer (GC). Our study evaluates POC clinical impact in the prognosis of patients underwent neoadjuvant chemotherapy (NAC) for locally advanced Gastric Cancer (LAGC).

**Methods:**

We enrolled 256 patients who underwent D2/D3 gastric resection in a curative way for LAGC. 50 patients underwent NAC according to FLOT scheme. Overall survival (OS) and Disease free survival (DFS) were investigated by univariate and multivariate analysis in NAC and non NAC patients.

**Results:**

There were 115 and 27 POC occurrences respectively in upfront surgery group and NAC one. 5 year-OS in upfront surgery patients was better in the arm without POC (59.1% vs. 52.1%), whereas 5 year-OS in NAC cohort was 81% and 79.7% for respectively patients with or without POC (*p* = 0.81, HR 1.196). 5y-OS strongly improved in patients with POC who underwent NAC rather than patients who underwent upfront surgery (81% vs 52.1%, respectively; *p* = 0.04, HR 2.186).

**Discussion:**

NAC remains the gold standard treatment for patients with locally advanced GC and it could be a positive prognostic factor for patients with POC. We demonstrated that the negative POC impact was reduced by positive NAC effect. Prospective and larger studies need to build exact evidence.

**Supplementary Information:**

The online version contains supplementary material available at 10.1007/s13304-025-02370-3.

## Introduction

Gastric cancer (GC) is the fifth most common cancer and the fifth leading cause of cancer death worldwide [1]. It is well known that surgery and chemotherapy are both fundamental in the management of GC. Since Magic Trial [2] was published, the treatment of locally advanced gastric cancer (LAGC) has terribly changed from an upfront surgery approach to a combination of peri-operative chemotherapy and surgery, apporting exciting results in terms of survival outcomes.

According to the International Guidelines, the standard surgical procedure for LAGC includes gastric resection combined to D2 lymphadenectomy [3]. Although this approach is standardized worldwide, the occurrence of post-operative complications (POC) ranges between 15 and 45% [4, 5].

It is well demonstrated that POC could negatively affect patients’ prognosis. The mechanism of their negative prognostic survival impact is unclear, even if some authors tried to explain it considering the important inflammatory response unleashed by POC [6]. At the same time, many studies have shown that patients undergone neoadjuvant chemotherapy (NAC) with POC showed a better prognosis compared to patients who underwent upfront surgery, as if NAC could protect the patient from POC negative impact [7].

POC are generally classified according to Clavien–Dindo Classification of surgical complications [8]. This classification is based on the therapy used to correct complication and consists of seven grades: grade I: Any deviation from the normal post-operative course without the need for pharmacological treatment or surgical, endoscopic, and radiological interventions; grade II: Requiring pharmacological treatment with drugs other than such allowed for grade I complications; grade III: Requiring surgical, endoscopic, or radiological intervention (IIIa: Intervention not under general anesthesia; IIIb: Intervention under general anesthesia); grade IV: Life-threatening complication requiring Intermediate Care/Intensive Care Unit Management (IVa: single-organ dysfunction (including dialysis); IVb: multi-organ dysfunction); and grade V: Death of a patient.

The literature is very poor and controversial about this topic. Thus, our aim is to investigate how post-surgical complications could influence LAGC patients prognosis focusing their prognostic survival influence in NAC patients.

## Materials and methods

### Patients

We evaluated a retrospective series of 256 patients affected by LAGC who underwent D2-or D2 plus total or subtotal gastrectomy from January 2004 to December 2023 in our Surgical Department at "Santa Maria della Misericordia” Hospital in Perugia (Italy).

Since 2007 a subgroup of them underwent pre-operative chemotherapy. We included patients with age ≥ 18 years, single primary gastric adenocarcinoma, with complete staging information and with a number of harvested lymph nodes ≥ 16.

We excluded patients who underwent surgery for Gastrointestinal Stromal Tumor (GIST), lymphoma, or other gastric neoplasia, those who had Siewert I–II carcinomas, those with specimen with micro- and macroscopic positive margin, underage patients, those who underwent palliative surgery, those with a number of harvested lymph nodes < 16, patients with incomplete data, and patients with grade V of Clavien–Dindo Classification.

The regimens of neoadjuvant treatment were DOX, ECF, FLOT, or FOLFOX chemotherapy as guidelines recommended [3]. Informed written consent was got by each patient before surgery.

All patients were followed up with physical examination, laboratory tests, and imaging every 3–6 months in the first year and then every 6–12 months for at least 5 years after surgery. All followed procedures were under the ethical standards of the responsible committee on human experimentation (institutional and national) and with the Helsinki Declaration of 1964 and later versions. All the patients signed a written consent to be included.

We defined the tumor stage according to the 8th edition of AJCC ypTNM staging system and we conducted an appropriate conversion in patients whose tumor stage was defined according to the previous TNM editions [9].

### Surgical procedure

All patients underwent total or distal gastrectomy associated to a D2 or D2 plus lymphadenectomy performing an open or mini-invasive approach. Distal gastrectomy was reconstructed according Billroth II or Roux‑en Y techniques, whereas during Total Gastrectomy we always used to perform a reconstruction according to Roux‑en Y technique. The anastomosis was made both mechanical and manual, intracorporeal or extracorporeal.

### Statistical analysis

Firstly, we evaluated the 5–10-year Overall Survival (OS) and 5–10-year Disease-Free Survival (DFS) of the whole population. We stratified patients according to the presence of POC analyzing their survival outcomes: 5–10-year OS and 5–10-year DFS. Successively, we stratified the entire population into two groups according to the performed treatment (upfront surgery vs. NAC plus surgery) and in each cohort we analyzed two subgroups of patients on the basis of the POC occurrence according to Clavien–Dindo Classification. If multiple complications occurred in a single patient, the highest grade was used.

Finally, we compared survival outcomes of patients in the two cohorts of patients showing the same grade of complications. All these parameters were retrospectively analyzed using a univariate analysis. Survival curves and disease survival rates were obtained using the Kaplan–Meier method and differences were compared using the log-rank test. Data management and statistical analyses were performed using Prism 10 Graph PAD software (Boston, MA, USA). A *p*-value < 0.05 was considered statistically significant.

## Results

Table 1 summarizes the main clinical and pathological features of the studied populations. The median age was 72 years old and the Male/Female (M/F) ratio was 1.6. The median follow-up was 85 months. The entire studied population was divided into two groups “Upfront” and “NAC.” In particular, 206 patients were categorized in the “Upfront” cohort and 50 patients in the “NAC” one.

**Table 1:** Clinical and pathological features of the studied population

	Upfront cohort (%) (*n* = 206)		NAC cohort (%) (*n* = 50)		Total (%) (*n* = 256)	
	nPOC (*n* = 91)	POC (*n* = 115)	nPOC (*n* = 27)	POC (n = 23)	nPOC (*n* = 118)	POC (*n* = 138)
Age, median (range)	74 (39–87)		65 (45–82)		71 (39–87)	
Sex						
Male	59 (64.8)	67 (58.3)	18 (66.6)	14 (60.9)	77 (65.3)	81 (58.7)
Female	32 (35.2)	48 (41.7)	9 (33.4)	9 (39.1)	41 (34.7)	57 (41.3)
Tumor location						
Upper	8 (8.8)	12 (10.4)	7 (25.9)	8 (34.8)	15 (12.7)	20 (14.5)
Middle	26 (28.6)	32 (27.8)	7 (25.9)	4 (17.4)	33 (27.9)	36 (26.1)
Lower	57 (62.6)	71 (61.8)	13 (48.2)	11 (47.8)	70 (59.4)	82 (59.4)
Histological type						
Intestinal	50 (54.9)	69 (60.0)	13 (48.2)	16 (69.7)	63 (53.4)	85 (61.6)
Diffuse	28 (30.8)	33 (28.7)	10 (37.0)	3 (13.0)	38 (32.2)	36 (26.1)
Mixed	13 (14.3)	13 (11.3)	4 (14.8)	4 (17.3)	17 (14.4)	17 (12.3)
Type of gastrectomy						
Total	29 (31.9)	42 (36.5)	13 (48.2)	14 (60.9)	42 (35.6)	56 (40.6)
Subtotal	62 (68.1)	73 (63.5)	14 (51.8)	9 (39.1)	76 (64.4)	82 (59.4)
Lymph node dissection						
D2	72 (79.1)	95 (82.6)	20 (74.1)	18 (78.3)	92 (77.9)	113 (81.9)
> D2	19 (20.9)	20 (17.4)	7 (25.9)	5 (21.7)	26 (22.1)	25 (18.1)
(y)pT						
(y)pT0	1 (1.0)	2 (1.7)	1 (3.6)	1 (4.3)	2 (1.7)	3 (2.2)
(y)pT1	26 (28.6)	20 (17.4)	4 (14.8)	3 (13.0)	30 (25.4)	23 (16.7)
(y)pT2	7 (7.7)	15 (13.1)	9 (33.4)	2 (8.7)	16 (13.6)	17 (12.3)
(y)pT3	32 (35.2)	36 (31.3)	9 (33.4)	10 (43.5)	41 (34.7)	46 (33.3)
(y)pT4	25 (27.5)	42 (36.5)	4 (14.8)	7 (30.5)	29 (24.6)	49 (35.5)
(y)pN						
(y)pN0	43 (47.3)	39 (33.9)	14 (51.9)	9 (39.1)	57 (48.3)	48 (34.8)
(y)pN1	6 (6.6)	18 (15.7)	3 (11.1)	5 (21.7)	9 (7.6)	23 (16.7)
(y)pN2	9 (9.8)	23 (20)	7 (25.9)	5 (21.7)	16 (13.6)	28 (20.3)
(y)pN3	33 (36.3)	35 (30.4)	3 (11.1)	4 (17.4)	36 (30.5)	39 (28.2)
(y)pM						
(y)pM0	76 (83.5)	94 (81.7)	27 (100)	21 (91.3)	103 (87.3)	115 (83.3)
(y)pM1	15 (16.5)	21 (18.3)	0 (0)	2 (8.7)	15 (12.7)	23 (16.7)
Pathological stage						
I	30 (32.9)	26 (22.6)	10 (37.0)	4 (17.3)	40 (33.8)	30 (21.7)
II	18 (19.8)	25 (21.7)	7 (25.9)	10 (43.5)	25 (21.2)	35 (25.4)
III	24 (26.4)	38 (33.1)	8 (29.6)	7 (30.5)	32 (27.2)	45 (32.6)
IV	19 (20.9)	26 (22.6)	2 (7.5)	2 (8.7)	21 (17.8)	28 (20.3)

**Fig. 1:**
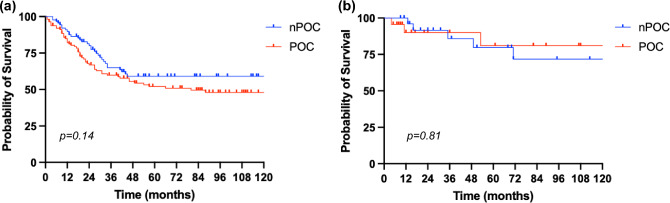
Overall Survival in upfront cohort (**a**) and NAC cohort (**b**)

The 5- and 10-y OS of the entire population were 59.2% and 56.4%, respectively (Supplementary Fig. 1). Instead, the 5- and 10-y DFS were 57% (supplementary Fig. 2). Figure 1 shows the 5–10-year OS of patients stratified according to the presence of POC, distinguished, respectively, in POC and nPOC (without post-operative complications) within the above mentioned two cohorts, “Upfront” and “NAC.” In the first cohort, the 5- and 10-year OS were 59.1% in the nPOC group and 52.1% and 47.9% in the POC group, respectively. The survival of the nPOC group was better than the POC group, but no statistically significant difference was found (*p* = 0.14).

We observed the same in the “NAC” cohort, where the 5- and 10-year OS were even better in POC group than in the nPOC group, 81% vs. 79.7% and 81% vs. 71.8%, respectively (p = 0.81).

**Fig. 2:**
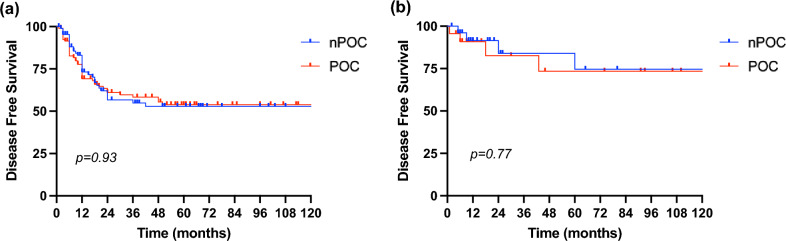
Disease Free Survival in upfront cohort (**a**) and NAC cohort (**b**)

Figure 2 describes the DFS of nPOC and POC groups in the two cohorts of patients. In "Upfront” cohort, the 5- and 10-y DFS were 52.8% in the nPOC group and 53.9% in the POC group. No statistically significant difference was found (*p* = 0.93). In the “NAC” cohort, patients without POC had a DFS close to that of patients with POC (74.6% vs. 73.4%, respectively; *p* = 0.77).

**Fig. 3:**
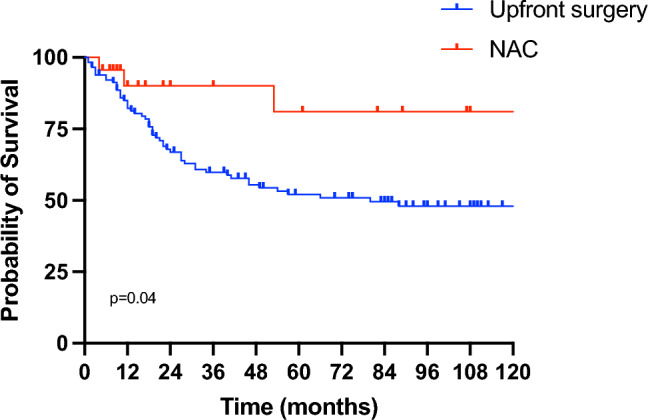
Overall Survival in patients with postoperative complications in the two cohorts

Finally, we only considered the POC group, dichotomizing patients in upfront and NAC. Comparing clinical and pathological features of the two subgroups of patients with POC, we showed no difference in sex, histological type, lymph node dissection, ypT, ypN, ypM, and pathological stage. Instead, the tumor location, the type of gastrectomy, and the grade of POC were different between the two groups (*p* = 0.009, *p* = 0.029, and *p* = 0.037, respectively) as well as are described in Table 2. After that, we analyzed survival and we showed a statistically significant difference in OS between patients with POC who underwent surgery at first or NAC before surgery (p = 0.04, HR 3.236, 95% CI 1.569–6.675) (Fig. 3). The first group had a 5- and 10-year OS of 52.1% and 47.9%, whereas the latter group had a 5- and 10-y OS of 81%. Considering DFS, we did not find a statistically significant difference between the two cohorts. In fact, the 5- and 10-year DFS of patients underwent surgery at first were both 53.9% and the 5- and 10-y DFS of patients underwent NAC were both 73.4% (*p* = 0.135; HR 2.110, 95% CI 0.974–4.565). Moreover, we found a statistically significant difference in OS in the POC group considering age (*p* = 0.02), NAC (*p* = 0.02), pT (*p* = 0.0005), pN (*p* < 0.0001), pM (*p* = 0.008), pStage (*p* < 0.0001), lymphovascular invasion (LVI) (*p* = 0.0008), and perineurial invasion (PNI) (*p* = 0.002). In multivariate analysis, age and NAC were identified as independent prognostic factors for OS (p = 0.0071 and p = 0.0098, respectively).

**Table 2:** Clinical and pathological features of patients with POC

	Upfront cohort (%) (*n* = 115)	NAC cohort (%) (*n* = 23)	*p* value	All patients (%) (*n* = 138)	Univariate analysis, *p* value
Sex			> 0.999		.667
Male	67 (58.3)	14 (60.9)		81 (58.7)	
Female	48 (41.7)	9 (39.1)		57 (41.3)	
Age			0.011		0.027
< 65	31 (27)	13 (56.5)			
≥ 65	84 (73)	10 (43.5)			
Tumor location			0.009		0.892
Upper	12 (10.4)	8 (34.8)		20 (14.5)	
Middle	32 (27.8)	4 (17.4)		36 (26.1)	
Lower	71 (61.8)	11 (47.8)		82 (59.4)	
Histological type			0.264		0.225
Intestinal	69 (60.0)	16 (69.6)		85 (61.6)	
Diffuse	33 (28.7)	3 (13.0)		36 (26.1)	
Mixed	13 (11.3)	4 (17.4)		17 (12.3)	
Type of gastrectomy			0.029		0.760
Total	42 (36.5)	14 (60.9)		56 (40.6)	
Subtotal	73 (63.5)	9 (39.1)		82 (59.4)	
Lymph node dissection			0.621		0.961
D2	95 (82.6)	18 (78.3)		113 (81.9)	
> D2	20 (17.4)	5 (21.7)		25 (18.1)	
(y)pT			0.705		0.0005
(y)pT0	2 (1.7)	1 (4.4)		3 (2.2)	
(y)pT1	20 (17.4)	3 (13.0)		23 (16.7)	
(y)pT2	15 (13.0)	2 (8.7)		17 (12.3)	
(y)pT3	36 (31.3)	10 (43.5)		46 (33.3)	
(y)pT4	42 (36.5)	7 (30.4)		49 (35.5)	
(y)pN			0.624		< 0.0001
(y)pN0	39 (33.9)	9 (39.1)		48 (34.8)	
(y)pN1	18 (15.7)	5 (21.7)		23 (16.7)	
(y)pN2	23 (20)	5 (21.7)		28 (20.3)	
(y)pN3	35 (30.4)	4 (17.4)		39 (28.2)	
(y)pM			0.261		0.008
(y)pM0	94 (81.7)	21 (91.3)		115 (83.3)	
(y)pM1	21 (18.3)	2 (8.7)		23 (16.7)	
Pathological stage			0.128		< 0.0001
I	26 (22.6)	4 (17.4)		30 (21.7)	
II	25 (21.7)	10 (43.5)		35 (25.4)	
III	38 (33.1)	7 (30.4)		45 (32.6)	
IV	26 (22.6)	2 (8.7)		28 (20.3)	
Clavien-Dindo			0.037		0.396
I	18 (15.7)	8 (34.8)		26 (18.9)	
II	81 (70.4)	9 (39.2)		90 (65.2)	
III	9 (7.8)	3 (13.0)		12 (8.7)	
IV	7 (6.1)	3 (13.0)		10 (7.2)

The severity and type of POC are summarized in Table 3. No grade V has been included in this study. Among the 256 patients, grade I, II, III, and IV complications were observed in 26 (10.2%), 90 (35.2%), 12 (4.7%), and 10 (3.9%) patients, respectively.

**Table 3:** Severity and type of POC

	Upfront cohort (%) (*n* = 115)	NAC cohort (%) (*n* = 23)	Total (%) (*n* = 138)
Grade I	*n* = 18	*n* = 8	*n* = 26
Anemia without transfusion	0	1 (4.3)	1 (0.7)
Pleural effusion	11 (9.6)	4 (17.4)	15 (10.9)
Wound infectious	2 (1.7)	1 (4.3)	3 (2.2)
Non infectious diarrhea	2 (1.7)	0	2 (1.4)
Vomiting	2 (1.7)	1 (4.3)	3 (2.2)
Urinary retention	1 (0.9)	1 (4.3)	2 (1.4)
Grade II	*n* = 81	*n* = 9	*n* = 90
Atrial fibrillation requiring pharmacological treatment	2 (1.7)	0	2 (1.4)
Anastomotic leakage requiring antibiotics	2 (1.7)	0	2 (1.4)
Abdominal abscess requiring antibiotics	5 (4.3)	1 (4.3)	6 (4.3)
Urinary tract infectious requiring antibiotics	3 (2.6)	0	3 (2.2)
Wound infectious requiring antibiotics	4 (3.5)	0	4 (2.9)
Pneumonia requiring antibiotics	16 (13.9)	3 (13)	19 (13.8)
Anemia requiring transfusion	49 (42.6)	5 (21.7)	54 (39.1)
Grade III	*n* = 9	*n* = 3	*n* = 12
Anastomotic leakage requiring surgery	3 (2.6)	3 (13)	6 (4.3)
Abdominal abscess requiring percutaneous drainage	4 (3.5)	0	4 (2.9)
Pleural effusion requiring drainage	1 (0.9)	0	1 (0.7)
Pancreatic fistula requiring drainage	1 (0.9)	0	1 (0.7)
Grade IV	*n* = 7	*n* = 3	*n* = 10
Lung failure	1 (0.9)	1 (4.3)	2 (1.4)
Heart failure	0	1 (4.3)	1 (0.7)
Anastomotic leakage	4 (3.5)	0	4 (2.9)
Abdominal abscess	1 (0.9)	1 (4.3)	2 (1.4)
Necrotizing pancreatitis	1 (0.9)	0	1 (0.7)

Analyzing the different grades of POC, we showed a higher number of grade I and II POC than grade III and IV (116 vs. 22, respectively). In addition, considering only grade II, we showed that the major cause of complications was anemia requiring transfusion.

Considering the “Upfront” cohort, we did not find a statistically significant difference in OS according to grades I–II and grades III–IV; in particular, patients with grade I–II had a 5- and 10-year OS of 54.1% and 49.6%, whereas the 5-year OS of patients with grades III–IV was 39.3% (*p* = 0.20). In the “NAC” group we did not find a statistically significant difference in 5-y OS (83.3% vs. 80.7%; *p* = 0.91). No significant statistically difference was found in DFS.

## Discussion

The risk of complications following treatment for esophagogastric malignancy is not insignificant and overall survival rates are negatively affected by them as many studies have well demonstrated [10, 11]. Yoo HM et al. described an incidence of post-operative complications after gastrectomy ranged from 6.7 to 35% and our incidence was about 40% considering the major complications [12, 13].

Post-operative complication is a significant prognostic factor that could cause the death of patient, that added to surgical stress could aggravate comorbidities and the frailty of GC patients. Our findings also reinforce this issue, so it is crucial to avoid POC not only for better early post-surgical outcomes but also to prolong patient survival.

Another aim of our study was to investigate whether the severity of POC is related to a worse prognosis. Analyzing our results we demonstrated that by increasing the severity of complications (grades III and IV), survival outcomes decreased. One possible explanation could be that the more severe complication is associated with a higher level of inflammatory cytokines and growth factors, that it could stimulate the growth of residual cancer cells by a protein synthesis in the liver in favor of pro-inflammatory leukins depressing the activity of T-cell lymphocytes.

This fact was more evident in the Upfront surgery group than in the NAC one, enforcing the concept that NAC could protect patients from the negativity brought by complications.

Comparing the clinical pathological characteristics between patients with and without complications, it was possible to see some differences in particular regarding the surgical procedure and intraoperative findings that require total gastrectomy. This surgery is usually performed for upper-third GC and it is associated with a longer time of operation, increased blood loss, and an increased incidence of POC.

Complications are generally associated with a longer hospital stay that affects the onset of adjuvant chemotherapy (AC). In addition, a worse physical condition of the patients would make it difficult to start adjuvant chemotherapy and the delay in starting AC is correlated with a significantly more difficult post-operative survival of patients with CG [14]. Therefore, NAC, which is not affected by the post-operative clinical course including complications, should be considered as the best treatment option for LAGC. It is fundamental to avoid POC after GC surgery but understand how is the real issue. Probably, the surgeon could improve post-surgical immune response adopting different measures, such as implementing mini-invasive approach or reducing intra- and post-operative blood transfusions which cause immune system dysfunction by increasing neoplastic cell transformation. Our study has some limitations being a retrospective analysis requiring a validation in an external center.

In conclusion, NAC may be a promising treatment option as recommended by the major guidelines; our results reinforced the concept that NAC influences the survival outcomes being an independent variable in complicated patients.

## Supplementary Information

Below is the link to the electronic supplementary material.

Supplementary file1 (PDF 52 KB)

## Data Availability

The datasets used and/or analyzed during the current study are available from the corresponding author on reasonable request.
