# Supplementary figures and images for: Survival prognostic impact of post-surgical complications in gastric cancer patients after neoadjuvant chemotherapy: a single western center analysis
Source: Updates Surg. 2025 Aug 19;78(2):557–64. doi: 10.1007/s13304-025-02370-3 (PMC13212410; doi:10.1007/s13304-025-02370-3)

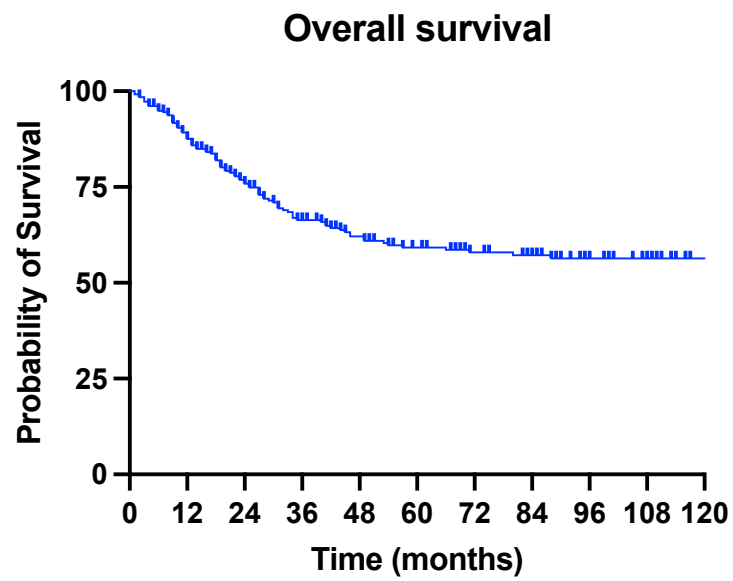

Supplementary Figure 1. Overall Survival

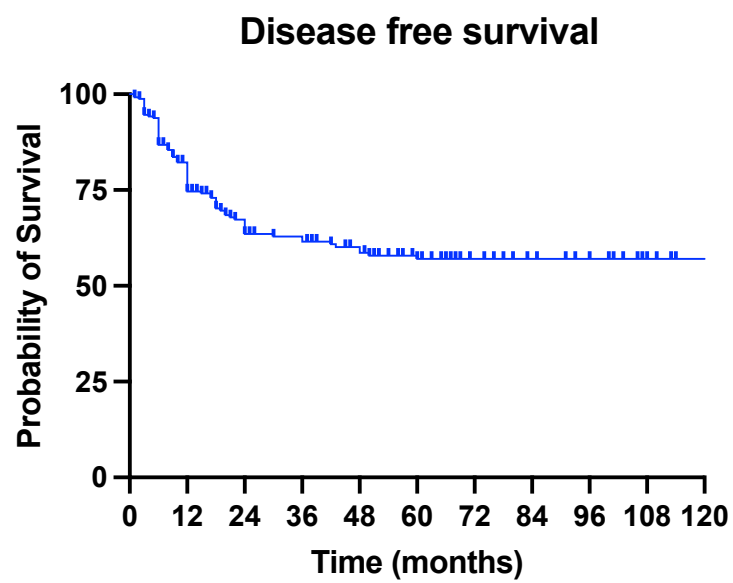

Supplementary Figure 2. Disease Free Survival

Supplement: Supplementary file 1 — Supplementary file1 (PDF 52 KB) [file 13304_2025_2370_MOESM1_ESM.pdf]
